# Aberrant somatic hypermutation of *CCND1* generates non-coding drivers of mantle cell lymphomagenesis

**DOI:** 10.1038/s41417-022-00428-7

**Published:** 2022-02-10

**Authors:** Heiko Müller, Wencke Walter, Stephan Hutter, Niroshan Nadarajah, Manja Meggendorfer, Wolfgang Kern, Torsten Haferlach, Claudia Haferlach

**Affiliations:** MLL Munich Leukemia Laboratory, Munich, Germany

**Keywords:** Lymphoma, Biomarkers

## Abstract

Aberrant somatic hypermutation (aSHM) can target proto-oncogenes and drive oncogenesis. In mantle cell lymphoma (MCL), *CCND1* is targeted by aSHM in the non-nodal subtype (nnMCL), giving rise to exon1 encoded mutant proteins like E36K, Y44D, and C47S that contribute to lymphomagenesis by virtue of their increased protein stability and nuclear localization. However, the vast majority of somatic variants generated by aSHM are found in the first intron of *CCND1* but their significance for mantle cell lymphomagenesis is unknown. We performed whole-genome and whole-transcriptome sequencing in 84 MCL patients to explore the contribution of non-coding somatic variants created by aSHM to lymphomagenesis. We show that non-coding variants are enriched in a MCL specific manner in transcription factor-binding sites, that non-coding variants are associated with increased *CCND1* mRNA expression, and that coding variants in the first exon of *CCND1* are more often synonymous or cause benign amino acid changes than in other types of lymphomas carrying a t(11;14) translocation. Therefore, the increased frequency of somatic variants due to aSHM might be a consequence of selection pressure manifested at the transcriptional level rather than being a mere mechanistic consequence of misguided activation-induced cytidine deaminase (AID) activity.

## Introduction

Mantle cell lymphoma (MCL) is an aggressive B cell neoplasm genetically characterized by the translocation t(11;14)(q13;q32), leading to *CCND1* overexpression [1, 2]. Two molecular subtypes are currently recognized: (1) Classical MCL (cMCL) is composed of B cells with minimally mutated or unmutated immunoglobulin heavy chain variable (IGHV) region that express *SOX11*. Patients have generalized lymphadenopathy and the outcome is adverse. (2) In non-nodal MCL (nnMCL), B cells do not express *SOX11* and often carry mutated IGHV. Here the involved organs are peripheral blood, bone marrow, and spleen. Cases are often clinically indolent [1]. The cells of origin are believed to be naive B cells that do not undergo germinal center reactions as in the case of cMCL and memory B cells in nnMCL [3].

Frequent molecular alterations in MCL are found in *ATM*, *TP53*, *NSD2*, *KMT2D*, *NOTCH1/2*, *UBR5*, *BIRC3*, *TRAF2*, *MAP2K14*, *CARD11*, *SMARCA4*, and *BTK* [3]. The most characteristic alteration in MCL is, however, rearrangement of the *CCND1* locus, which leads to the juxtaposition of the strong immunoglobulin heavy-chain enhancer on chr14, and results in *CCND1* overexpression [4, 5]. The *CCND1* transcript can also be stabilized by deletions or point mutations in the 3’UTR eliminating miRNA binding sites or creating premature polyadenylation sites [6, 7]. Stabilization of CCND1 at the protein level has also been observed in MCL. Alternatively, spliced isoforms lacking the T286 phosphorylation site needed for CCND1 degradation [8–10] and amino acid changes linked to increased protein stability have been described [11].

Widespread occurrence of *CCND1* mutations caused by aberrant somatic hypermutation has first been detected by [12] using whole-transcriptome sequencing (WTS). Other screens have confirmed these findings [13–15]. However, the biological significance of these mutations has not been fully clarified. One reason may be found in the mechanism of aberrant somatic hypermutation (aSHM) generation by activation-induced cytidine deaminase (AID), whose activity is tightly regulated and restricted to 1–2 kb from the transcriptional start site of its target genes [16]. Another reason may be that the effects of aSHM are still incompletely understood.

Here we present the analysis of the mutation spectrum of 84 MCL patients whose genomes and transcriptomes have been fully sequenced. We report that *CCND1* mutations generated by aSHM are particularly frequently observed in MCL as compared to other lymphomas carrying the t(11;14)(q13;q32) translocation. Our results are compatible with the hypothesis that *CCND1* mRNA expression levels are a rate-limiting factor for MCL lymphomagenesis and that non-coding mutations generated by aSHM are selected for their impact on *CCND1* mRNA expression.

## Materials and methods

### Patients and samples

The cohort includes 84 patients diagnosed with MCL among 4610 samples (5k data set) from a wide variety of hematological malignancies (Table 1 and Supplementary Table 1). The selection of samples for the 5k data set was based on the following criteria:

1. Consent of patients for research use of their data and good prospects for collaborative follow-up studies with clinicians.
2. Uniform coverage of molecular subtypes of master cohorts as defined by WHO [2] criteria.
3. Percentage of aberrant white blood cells at least 30%.

**Table 1:** Overview of samples analyzed by whole-genome sequencing (WGS).

Abbreviation	Description	Samples	Median age	Female/male
aCML	Atypical chronic myeloid leukemia	77	74	25/52
AML	Acute myeloid leukemia	750	68	344/406
AUL	Acute undifferentiated leukemia	37	76	14/23
BCP-ALL	B cell precursor acute lymphoblastic leukemia	285	54	137/148
B-NHL	B cell non-Hodgkin’s lymphoma	59	71	25/34
BPDCN	Blastic plasmacytoid dendritic cell neoplasm	24	74	1/23
CLL	Chronic lymphocytic leukemia	306	67	110/196
CML	Chronic myeloid leukemia	110	57	47/63
CMML	Chronic myelomonocytic leukemia	208	77	69/139
FL	Follicular lymphoma	64	54	32/32
HCL	Hairy cell leukemia	92	64	19/73
HGBL	High-grade B cell lymphoma	65	70	33/32
LPL	Lymphoplasmacytic lymphoma	61	71	14/47
MC	Mastocytosis	117	57	51/66
MCL	Mantle cell lymphoma	84	69	29/55
MDS	Myelodysplastic syndrome	682	73	291/391
MDS/MPN-RS-T	Myelodysplastic syndrome/myeloproliferative neoplasm with ring sideroblasts and thrombocytosis	87	74	51/36
MDS/MPN-U	Myelodysplastic/myeloproliferative neoplasms, unclassifiable	87	75	35/52
MGUS	IgM monoclonal gammopathy of undetermined significance	20	61	5/15
MLN_eo	Myeloid or lymphoid neoplasms associated with eosinophilia	47	52	6/41
MM	Multiple myeloma	358	67	159/199
MPAL	Mixed-phenotype acute leukemia	38	64	16/22
MPN	Myeloproliferative neoplasm	355	68	146/209
MZL	Marginal zone lymphoma	80	71	33/47
NK	Natural killer cell neoplasm	110	69	44/66
PNH	Paroxysmal nocturnal hemoglobinuria	117	43	67/50
PPBL	Polyclonal B cell lymphocytosis	47	49	37/10
T-ALL	T cell acute lymphoblastic leukemia	121	37	37/84
T-NHL	T cell non-Hodgkin’s lymphoma	94	69	41/53
vHCL	Hairy cell leukemia variant	28	80	12/16

Bone marrow (BM), and peripheral blood (PB) samples from patients had been sent to MLL Leukemia Laboratory between 2006 and 2020 for immediate diagnostic work-up. The respective diagnosis was established based on cytomorphology, immunophenotyping, cytogenetics, fluorescence in situ hybridization (FISH), and molecular genetics following WHO guidelines [2]. All patients gave their written informed consent for scientific evaluations. The study was approved by the Internal Review Board and adhered to the tenets of the Declaration of Helsinki. DNA samples from BM and/or PB, at diagnosis or before treatment, were collected from all cases and DNA and total RNA extracted using the MagNA Pure 96 Instrument and the MagNAPure96 DNA and Viral NA LV Kit and MagNA Pure 96 Cellular RNA LV Kit, respectively (Roche LifeScience, Mannheim, Germany) [17].

### Whole-genome sequencing (WGS) and analysis

WGS libraries were prepared from 1 µg of DNA with the TruSeq PCR free library prep kit following the manufacturer’s recommendations (Illumina, San Diego, CA, USA) and 2×150 bp paired-end sequences were generated on a NovaSeq 6000 or HiSeqX instrument with 100× coverage (Illumina, San Diego, CA, USA) and processed as described previously [17, 18]. A so-called Tumor/Unmatched normal workflow was used for variant calling to reduce technical artifacts and germline calls. Each variant was queried against the gnomAD database (v2.1.1) and variants with global population frequencies >0.5% were excluded. The final analysis was performed only on PASS filtered variants. Mutational signatures were analyzed using the R package MutSignatures [19]. Oncoprint representations of mutation data were generated using the R package ComplexHeatmap [20].

### WTS and analysis

For transcriptome analysis, the TruSeq Total Stranded RNA kit was used, starting with 250 ng of total RNA, to generate RNA libraries following the manufacturer’s recommendations (Illumina, San Diego, CA, USA). In all, 2×100 bp paired-end reads were sequenced on the NovaSeq 6000 with a median of 50 million reads per sample (Illumina, San Diego, CA, USA) and processed as described previously [17]. Counts were extracted with Cufflinks (v2.2.1) [21]. Counts were normalized applying the variance stabilizing transformation normalization method. Genes were kept if they were expressed (>5 CPM) in at least 66% of the samples. Gene expression differences were assessed using the DESeq2 package [22]. Model parameters included sequencing run, aSHM mutation status, material (peripheral blood or bone marrow), gender, chr11 breakpoint distance from *CCND1* TSS, and mutation status of *ATM*, *KMT2D*, *NSD2*, and *TP53*. The significance of coefficients in a negative binomial generalized linear model was estimated using the Wald test. Gene set enrichment analysis was performed with the GSEA package [23, 24] using a *CCND1* pre-ranked list based on DESeq2 normalized expression counts. Cluster analysis was carried out using the R package pheatmap (https://github.com/raivokolde/pheatmap).

### Variant enrichment analysis

Enrichment analysis using bipartite graph models was performed essentially as described [25, 26]. Briefly, bipartite graph models consisting of variant bins on one side of the graph and samples on the other side were constructed. The degree of bin vertices in this graph reflects the number of variants in that bin. Randomization of the graph yields a statistical null model. Two subgraphs were then constructed: one containing only MCL samples and the other containing only non-MCL samples. The significance of deviation of bin vertex degrees in the subgraphs from the predictions of the null model is then estimated using Poisson-binomial *Z*-scores and the bi-binomial approximation [26] of Poisson binomial *P* values.

### Motif searches

Motif searches were performed using the AME and the Centrimo apps of the MEME suite [27] and by motif consensus matching to motifs listed in Jaspar [28] and HOCOMOCO [29]. Motif matching results for all variants using wild-type and mutated sequences with 15 bases of padding to the left and to the right of the mutated position are listed in Supplementary Table 1.

### IGHV mutation status

IGHV mutation status was analyzed by Sanger sequencing of corresponding PCR fragments, by IGCaller software [30] analysis of WGS data, or both.

### In silico prediction of pathogenicity

The pathogenicity of amino acid changes was estimated using HePPY (10.1182/blood-2019-128488).

## Results

### The landscape of coding and non-coding variants in MCL

Our cohort of MCL patients comprises 84 cases. We performed enrichment analysis for the number of variants in bins of 10, 100, 1000, and 10,000 bases to identify genomic regions that carry significantly more variants in MCL patients as compared to our cohort of 4610 cases of leukemia and lymphoma (30 different entities, 5k data set, details listed in Table 1 and Supplementary Table 1) with whole-genome sequence data. We analyzed coding and non-coding variants separately.

**Fig. 1:**
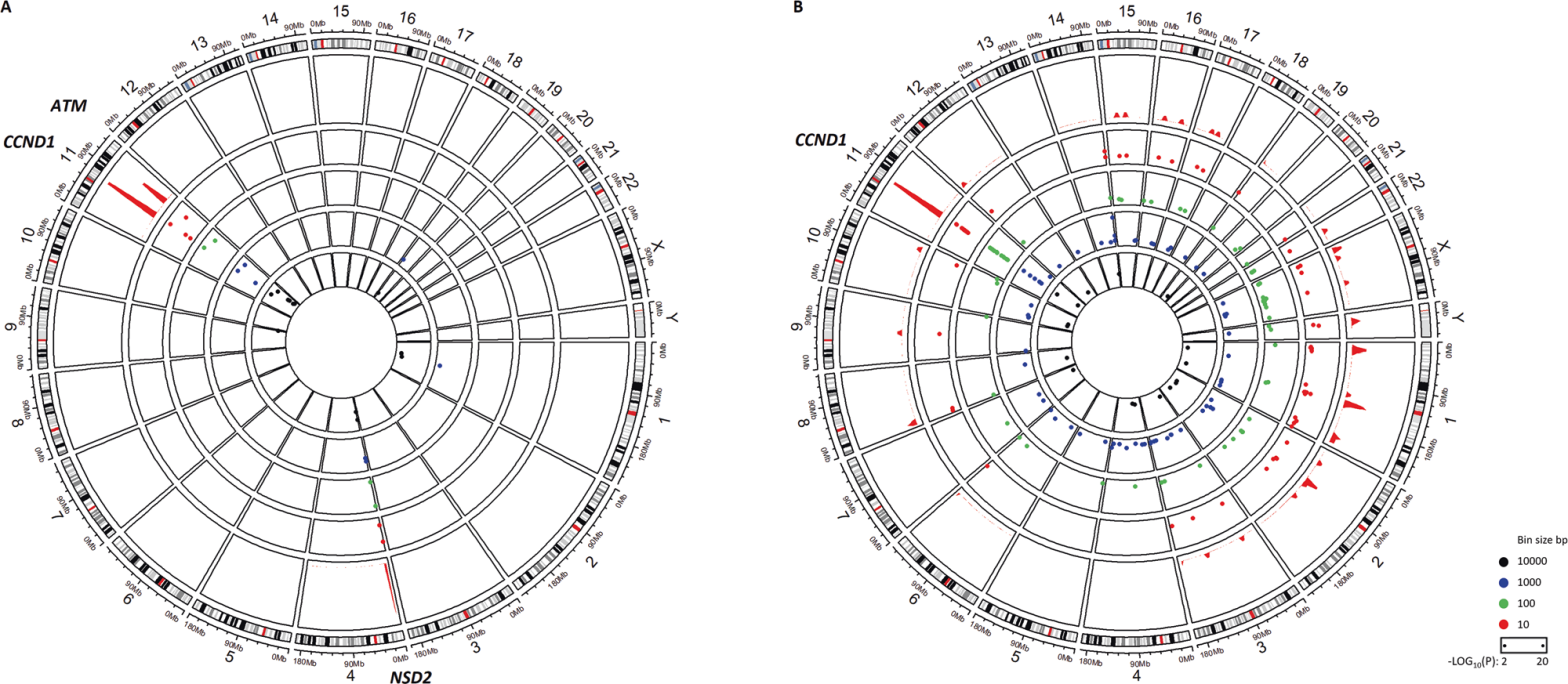
Genome-wide enrichment analysis for coding and non-coding variants in MCL. Circos plot of negative log_10_(*P*) for the number of variants in bins of different sizes. High values indicate hotspots of somatic mutation specific for MCL. The outer circle represents a genomic density plot of somatic mutations in the 10 bp bin (red). **A** Enrichment analysis for coding variants. **B** Enrichment analysis for non-coding variants.

Figure 1A shows the results for the analysis of coding variants. We detected three highly significant (−log_10_(*P*) ≥ 10) regions corresponding to *CCND1*, *ATM*, and *NSD2*. Coding mutations in these genes have been reported before for MCL [2] and this result validates our general approach. It is worth noting that our analysis is relative to other cohorts in our 5k data set (Table 1). Thus, genes like *TP53* and *KMT2D*, although mutated not only in MCL but also in many other malignancies, will not score as enriched in MCL in this analysis. Mutation data for *ATM*, *NSD2*, *KMT2D*, and *TP53* for all cohorts are listed in Supplementary Table 1.

The enrichment analysis for non-coding variants is shown in Fig. 1B. The complete list of genomic regions with significantly elevated densities of non-coding mutations in MCL can be found in Supplementary Table 2. The most significant region is found on chromosome 11 and corresponds to the *CCND1* locus. We therefore turned our attention to the *CCND1* locus for a more detailed analysis.

### MCL samples are prone to mutagenesis in the *CCND1* transcription regulatory region

**Fig. 2:**
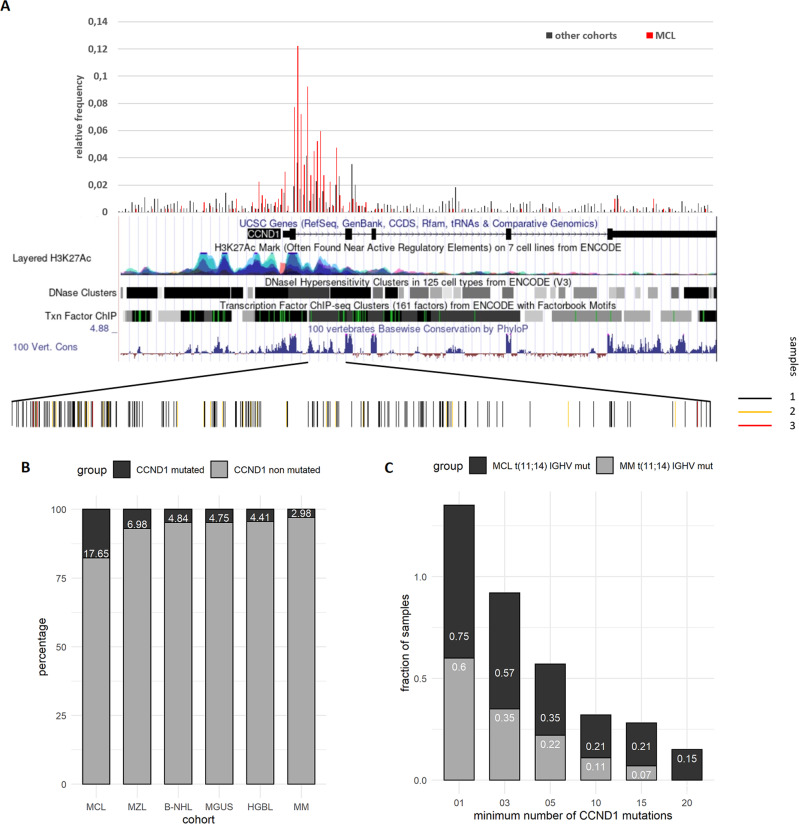
Frequency of non-coding variants in the *CCND1* locus. **A** Non-coding variants in the genomic region chr11:69450872-69469242 (hg19) covering the *CCND1* locus including 5 kb upstream of the TSS were collected for all MCL and non-MCL samples. Red bars indicate the relative frequency of non-coding variants in the MCL cohort. Black bars refer to the relative frequency of non-coding variants in non-MCL samples. A screenshot from the UCSC genome browser of the *CCND1* locus shows the density of H3K27 acetylation marks, the density of DNase 1 hypersensitivity sites, and transcription factor-binding regions, as well as the level of sequence conservation. The bars at the bottom of the panel indicate the distribution of individual variants in the first intron. The color of the bars indicates the number of samples a particular variant was observed in. **B** Plot of the percentage of samples with five or more non-coding variants in the *CCND1* transcription regulatory region (chr11: 69450872-69458025, hg19) for indicated cohorts. Abbreviations for cohort names are described in Table 1. **C** Plot of the fraction of samples by the minimum number of non-coding variants in the *CCND1* transcription regulatory region for Multiple Myeloma (MM) and MCL samples. Only samples carrying a t(11;14)(q13;q32) translocation and mutated *IGHV* status were included.

We generated histograms for the number of non-coding variants in 100 bp bins covering the entire *CCND1* locus and 5 kb upstream of the TSS (chr11:69455872, Fig. 2A). In this work, we refer to the 5 kb region upstream of the TSS as promoter region [31] whereas the region chr11:69450872-69458025 (hg19) encompassing the promoter, exon 1, and parts of intron 1 of CCND1 is denoted as transcription regulatory region. We noticed a strong increase of non-coding variants in the promoter region and the first intron in MCL samples as compared to other cohorts. The UCSC genome browser annotations of these regions show high levels of H3K27 acetylation, DNase 1 hypersensitivity, and transcription factor-binding sites (TFBSs). The bar plot at the bottom of Fig. 2A also shows that the variants in the first intron of the *CCND1* gene are not uniformly distributed. Rather, they appear to be clustered in some regions while others remain essentially void of variants.

The accumulation of variants in the promoter, the first exon, and the first intron of *CCND1* is not entirely specific for MCL. It is assumed that they are a byproduct of somatic hypermutation occurring in the germinal center microenvironment [12]. We detected such mutations in other B cell-derived malignancies such as multiple myeloma, albeit at a less pronounced level (see below).

However, MCL seems to be particularly prone to accumulate mutations in these regions. Figure 2B shows the percentage of samples with at least five non-coding mutations in the CCND1 transcription regulatory region for cohorts where such mutations were detected. The percentage of CCND1 mutated samples in the MCL cohort is two to three times higher than in other cohorts. The majority of these variants is found in intron 1 (Fig. 2A and Supplementary Table 2).

The nearly universal presence of the t(11;14)(q13;q32) translocation in MCL might be a confounding factor when comparing the percentage of *CCND1* mutated samples between different types of lymphoma. Therefore, we analyzed only samples carrying a t(11;14)(q13;q32) translocation. Only samples with mutated *IGHV* were included to ensure that SHM has taken place. We found 28 MCL samples and 45 multiple myeloma samples satisfying these criteria. Also in this more stringent comparison, MCL samples consistently carry more than twice as many non-coding variants in the *CCND1* locus as multiple myeloma samples (Fig. 2C). The majority of these variants are found in MCL samples with mutated *IGHV*, as can be seen from the data in Supplementary Table S1. MCL samples with mutated *IGHV* harbor nearly six times as many non-coding mutations than samples with unmutated *IGHV*. We conclude that MCL samples are particularly prone to accumulate non-coding variants in the regulatory region of the *CCND1* locus.

### Mutational signature analysis

The higher propensity of MCL for mutations in the *CCND1* locus might originate from differences in the mutational processes generating these mutations. We performed mutational signature analysis on mutations in the *CCND1* locus to address this question. We included all MCL samples in this analysis. For comparison, we used samples carrying the t(11;14)(q13;q32) translocation. We refer to this artificial cohort as non-MCL. It is mainly composed of multiple myeloma samples (45) and some samples from our marginal zone lymphoma (12), IgM monoclonal gammopathy of undetermined significance (3), and B cell non-Hodgkin lymphoma (5) cohorts.

**Fig. 3:**
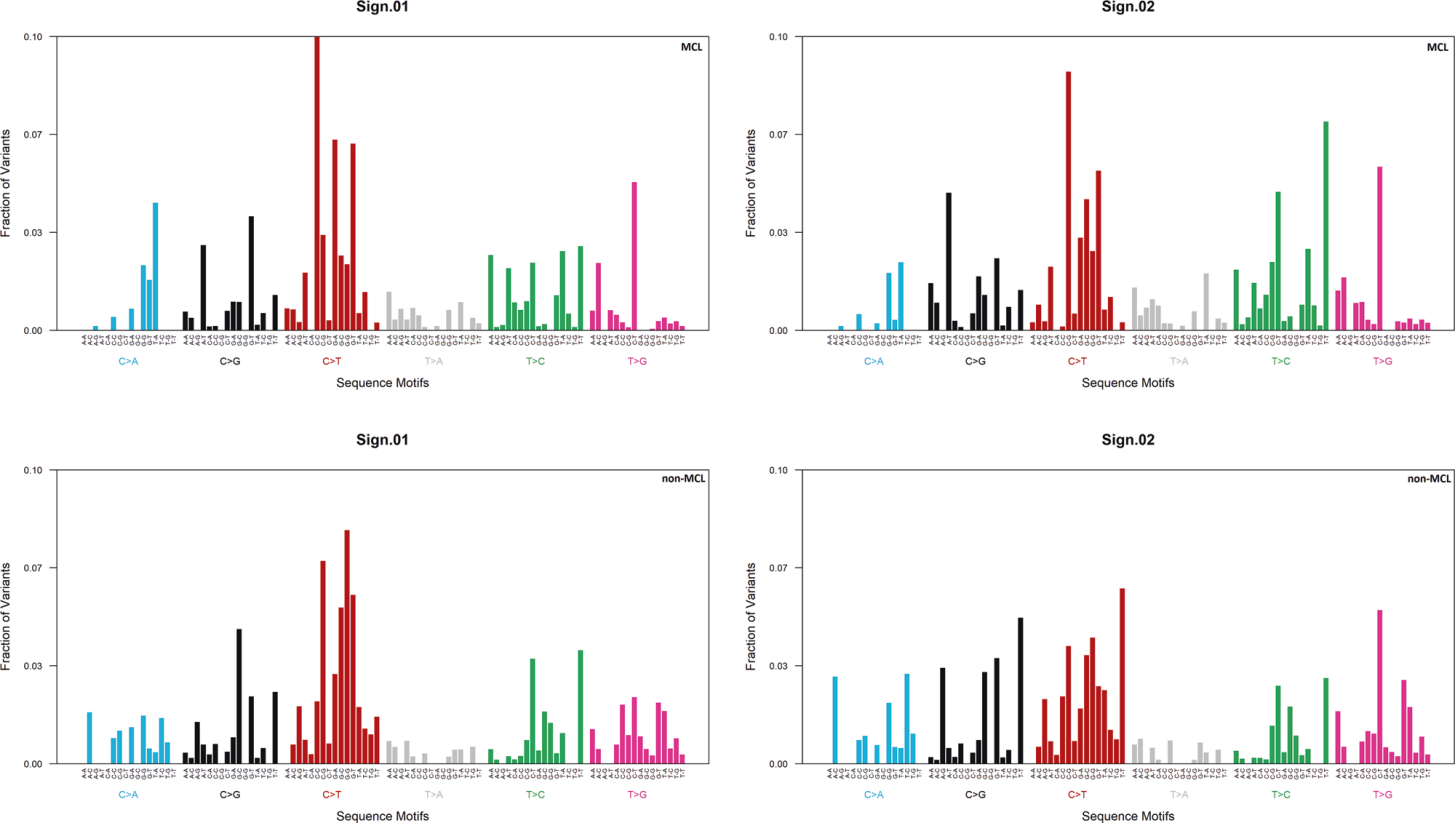
Mutational signature analysis for non-coding variants in the *CCND1* locus. Non-negative matrix factorization was applied to extract mutational signatures from MCL and non-MCL samples.

Figure 3 shows the results of extracting two mutational signatures by non-negative matrix factorization from the MCL and the non-MCL cohorts. The domineering type of mutation is a C-T transition in both cohorts. This observation is compatible with the activity of AID during somatic hypermutation. Comparison of our signatures to known COSMIC signatures [32] confirms that the mutational processes at work in both cohorts are largely similar (Table 2). We conclude that differences in mutational processes are unlikely to account for a large number of mutations in the *CCND1* locus in MCL.

**Table 2:** Mutational signatures were compared to known signatures compiled by COSMIC [32] using cosine similarity plots.

SBS	MCL	non-MCL	Description
sbs1		x	Spontaneous deamination of 5-methylcytosine (clock-like signature)
sbs3	x	x	Defective homologous recombination DNA damage repair
sbs5	x	x	unknown (clock-like signature)
sbs6	x	x	Defective DNA mismatch repair
sbs9	x	x	Polymerase eta somatic hypermutation activity
sbs15		x	Defective DNA mismatch repair
sbs23	x		Unknown
sbs31	x		Platinum chemotherapy treatment
sbs39	x	x	Unknown
sbs40	x	x	Unknown
sbs42	x	x	Haloalkane exposure
sbs44	x	x	Defective DNA mismatch repair
sbs84	x	x	Activity of activation-induced cytidine deaminase (AID)
sbs87		x	Thiopurine chemotherapy treatment

Signatures with cosine similarity values above the background are shown and marked with “x” depending on the cohort where they were detected.

### Cohort-specific enrichment of variant clusters

**Fig. 4:**
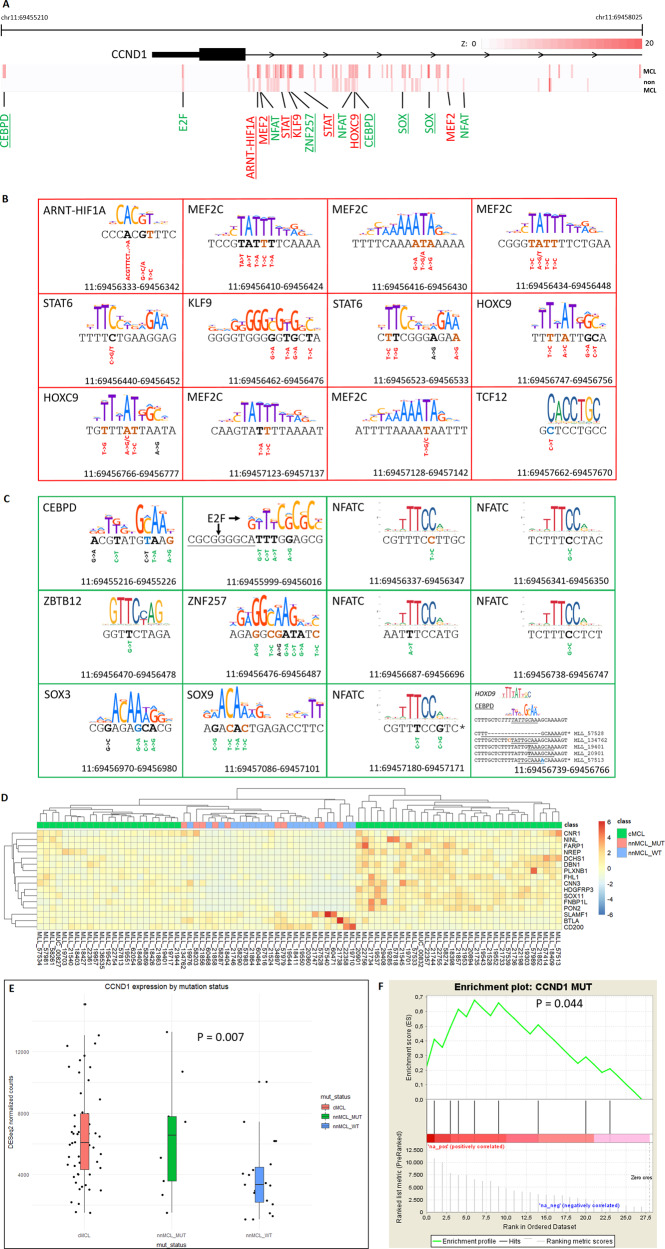
MCL-specific enrichment of non-coding variants and their impact on transcriptional regulation of *CCND1*. **A** Enrichment analysis of non-coding variants in the transcription regulatory region of the *CCND1* locus. Analysis was performed using bins of size 5–10 bp. The maximum enrichment *Z*-score for the number of variants per bin is plotted for each base on a color scale for the MCL and the non-MCL samples. Motif searches were performed using either wild-type or mutated cluster sequence. Best matching motifs are indicated in green or red letters. Red letters indicate motif matches to the wild-type sequence. Green letters indicate motif matches to the mutated sequence. MCL-specific enrichment of clusters is marked by underlining the name of the matching motif. **B**, **C** Motif matches to enriched clusters. For each cluster, the cluster sequence and the best matching motif sequence logo are shown. Mutated bases are shown in bold. Brown and blue sequence letters indicate mutations matching c-AID and nc-AID sequence context, respectively. Genomic positions refer to hg19 coordinates. Motif matches to the wild-type cluster sequence are shown with red framing (**B**) indicating that the cluster is associated with destructive mutations. Green framing indicates that the cluster is subject to constructive mutations (**C**). Base changes for individual variants are shown underneath the sequence, in red for destructive mutations, in green for constructive mutations, and in black for neutral mutations with regard to the quality of the motif match. Reverse complemented genomic sequence is marked with a “*”. **D** Cluster analysis using the gene signature identified by [34] to classify MCL samples as cMCL or nnMCL. Gene expression levels are based on DESeq2 normalized counts. The criteria to further subclassify nnMCL samples as nnMCL_MUT or nnMCL_WT are described in the main text. **E** Wald test for differential expression of *CCND1* mRNA depending on the mutation status of the transcription regulatory region of *CCND1*. **F** Gene set enrichment analysis for the mutation status of the transcription regulatory region of *CCND1*. A pre-ranked nnMCL sample list based on *CCND1* expression levels as measured by DESeq2 normalized counts was used. Black bars indicate the rank of nnMCL_MUT samples.

Next, we addressed the question of whether the clustered appearance of variants bears cohort specificity, which might be a reflection of an underlying biological significance. We performed enrichment analysis for the number of non-coding variants in bins from size five to ten base pairs using bipartite graph models (see “Materials and methods” section for details) in the region covering the *CCND1* promoter and the first intron. This analysis yields *Z*-score estimates for the significance of enrichment of variants in each bin for the MCL and the non-MCL cohorts. For each base position, the maximum *Z*-score was determined and plotted using a color scale. Figure 4A shows the results of this analysis. Some variant clusters are enriched in both the MCL and the non-MCL cohorts. However, the majority of clusters appear to display MCL cohort-specific enrichment.

We used motif searches to investigate whether the variant clusters co-localize with TFBS. The effect of mutations in TFBSs can be twofold: Some might serve to destabilize factor binding to a given site (destructive mutation) while others might change the binding specificity or increase the affinity of sites for some factors (constructive mutation). Constructive mutations are harder to link to a specific transcription factor as both the optimal binding motif and the factor best suited to bind to that site are not known. However, if an enriched cluster is subject to constructive mutations, the collection of mutations observed in that cluster should be a reflection of the unknown motif. Therefore, we used the mutations observed for each cluster for the construction of a set of mutated cluster sequences. Thus, for each cluster, the wild type and the mutated sequences were used for motif searches. Furthermore, for each variant, the sequence context was analyzed for matches compatible with classical AID (c-AID, C to T/G at WRCY motifs) and non-classical AID (nc-AID, A to C/G at WA motifs) mediated mutagenesis [33] (Supplementary Table 1). Both the wild type as well as the mutated sequence of each variant padded with 15 bases to the left and to the right were subjected to motif searches individually. The results are shown in Fig. 4B, C. We observed constructive mutations for NFAT, CEBP, E2F, and SOX motifs. MEF2, STAT, and HOX motifs were associated with destructive mutations. Interestingly, some motifs were identified more than once in different locations with a consistent preference for destructive or constructive mutations.

Next, we asked whether the enrichment of mutations in MCL-specific clusters had an impact on *CCND1* expression levels. To exclude confounding effects from cMCL cases, we used the gene signature identified by [34] to classify samples as either cMCL or nnMCL as shown in Fig. 4D. Twenty-eight samples were identified as nnMCL. nnMCL samples with at least two mutations in MCL-specific TFBS mutation clusters and 10 or more non-coding mutations in the CCND1 regulatory region were assigned to the mutated group (nnMCL_MUT). The remaining nnMCL samples were assigned to the wild-type group (nnMCL_WT). Next, we used whole transcriptome data for two types of analysis: First, we tested for differential expression of *CCND1* in the set of cMCL, nnMCL_MUT, and nnMCL_WT samples and, second, we performed gene set enrichment analysis using a list of nnMCL samples ranked according to *CCND1* expression level. The results of these tests are shown in Fig. 4E, F. We observed that nnMCL_MUT samples carrying mutations in the transcription regulatory region of *CCND1* have higher levels of *CCND1* mRNA than nnMCL_WT samples.

### Mutual exclusivity of *ATM* and *CCND1* mutations

**Fig. 5:**
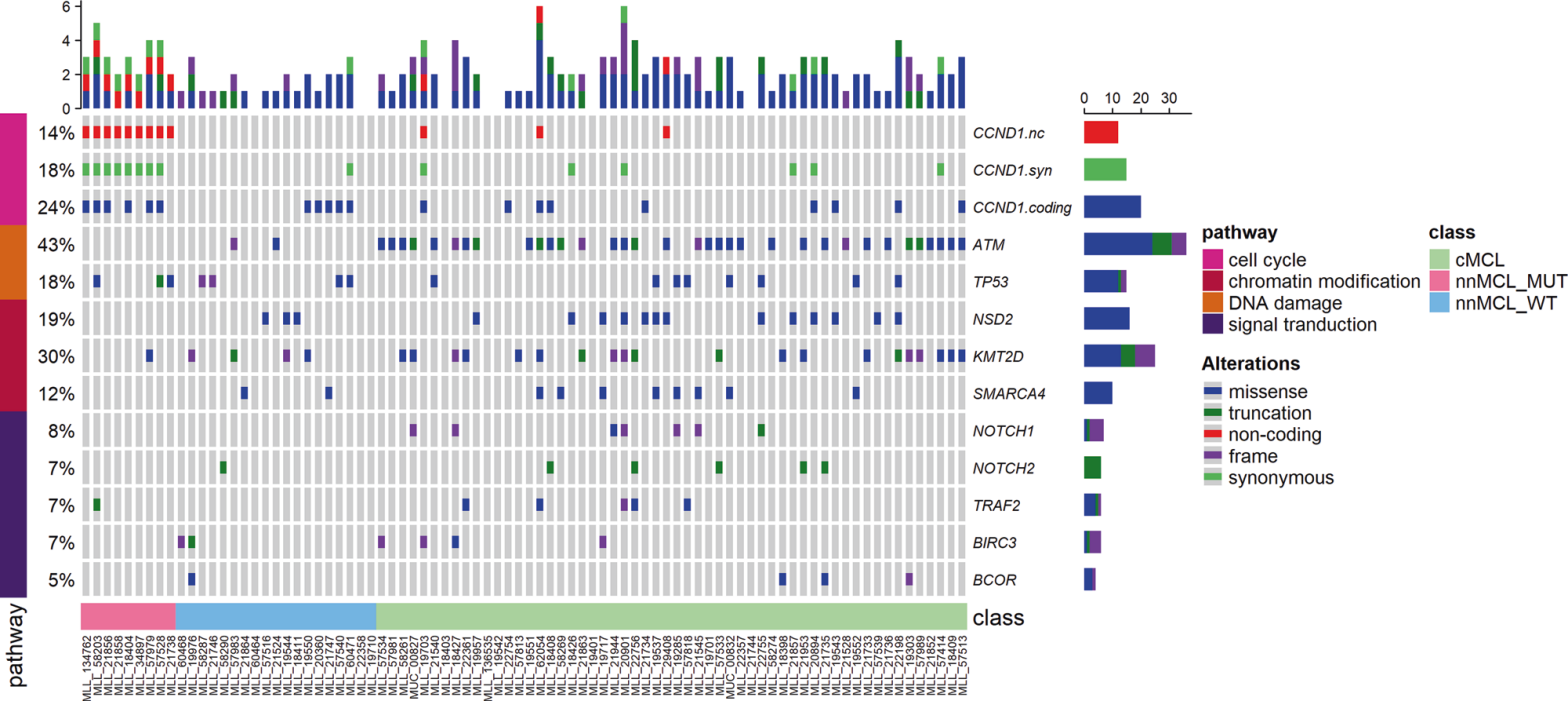
Mutual exclusivity of *ATM* and *CCND1* mutations. Oncoprint representation of selected genes found mutated in MCL and *CCND1* mutations. Mutated genes are depicted in rows, and cases are displayed in columns. Mutual exclusivity of *ATM* mutations and *CCND1* coding (missense) (*P* = 0.003), non-coding (*P* = 0.044), and synonymous (*P* = 0.009) mutations was detected (Fisher’s exact test).

We investigated the relationship between *CCND1* mutations and mutation patterns of known and potential driver genes in MCL with Oncoprint [35] (Fig. 5). Non-coding, missense, and synonymous *CCND1* mutations were analyzed separately. All types of *CCND1* mutations are found mainly in samples without mutations in *ATM* (*P* < 0.044, Fisher’s exact test). While it is a possibility that mutual exclusivity between *ATM* and *CCND1* mutations is based on biological differences in the cell of origin giving rise cMCL (naive B cells) and nnMCL (memory B cells), we did detect *ATM* mutations also in nnMCL cases. Therefore, this mutation pattern may be a reflection of a common endpoint of *ATM* and *CCND1* mutations, namely increased levels of CCND1 activity as ATM directly activates FBXO31 needed for CCND1 degradation [36].

### The abundance of synonymous or benign amino acid changes in *CCND1* exon1

**Fig. 6:**
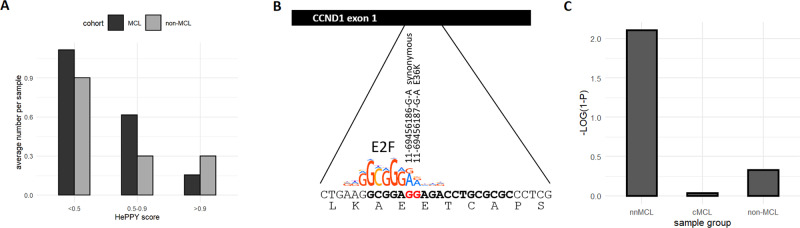
Prevalence of synonymous and benign mutations in the first exon of *CCND1* in MCL. **A** The number of samples with mutations in exon1 of *CCND1* by HePPY score is shown. HePPY is a predictor of missense variant pathogenicity (10.1182/blood-2019-128488). Low HePPY scores indicate benign amino acid changes. Deleterious mutations are associated with HePPY scores close to 1. **B** The sequence coding for CCND1 L32 to S41 is shown. Two frequently observed mutations in the first exon of *CCND1* are overlapping an E2F-binding site. The red guanine bases are often changed to adenine, which favors E2F binding. **C** Significance of the proportion of synonymous mutations in the sample groups displayed on the *x*-axis. *P* values were calculated using the cumulative hypergeometric distribution. The negative decadic logarithm of (1−*P*) is shown. Values >2 are considered significant.

As a further test of the hypothesis that aSHM generated mutations of the *CCND1* locus are driving *CCND1* mRNA expression levels and that CCND1 is a rate-limiting factor for MCL lymphomagenesis, we analyzed the spectrum of coding mutations in the first exon of *CCND1*. The first exon as part of the *CCND1* transcription regulatory region is potentially subject to selection pressure at the level of transcription as well as protein function. If protein function plays a crucial role, one would expect a mutation spectrum that is biased against synonymous mutations and mutations coding for benign amino acid changes. However, our results shown in Fig. 6A indicate that, in MCL, synonymous and benign mutations with low pathogenicity scores as estimated by HePPY (10.1182/blood-2019-128488) are more abundant as compared to non-MCL samples, where deleterious mutations with high HePPY scores prevail.

Interestingly, among the most frequently occurring mutations are 11-69456186-G-A and 11-69456187-G-A. As can be seen in Fig. 6B, both mutations stabilize an E2F-binding site, and in three out of eight samples carrying these mutations, they co-occur (Supplementary Table 3). 11-69456186-G-A is a synonymous mutation. 11-69456187-G-A codes for the previously reported E36K mutation [11]. It is possible that E36K adds a selective advantage to cells carrying this change. However, the frequent occurrence of the nearby synonymous mutation supports the hypothesis that the efficiency of transcription of the *CCND1* gene also plays a role. This notion is further supported by the significant enrichment of synonymous mutations observed in nnMCL samples as compared to cMCL and non-MCL samples shown in Fig. 6C.

## Discussion

We performed a genome-wide survey assessing the prevalence of non-coding mutations in MCL as compared to other types of leukemia and lymphoma. The *CCND1* locus emerges as the major hotspot of non-coding mutagenesis. MCL is characterized by a near-universal presence of a t(11;14)(q13;q32) translocation bringing the *CCND1* locus into close proximity with the immunoglobulin heavy-chain enhancer [4, 5], which can drive SHM in a subset of MCL cases [37]. SHM can occur outside of Ig loci. The first examples of SHM at non-physiological targets (aSHM) with a proven impact on target gene expression were *MYC*, *BCL6*, and *CD95* [38–40]. aSHM at the *CCND1* locus in MCL has first been reported by [12]. However, the restriction of AID activity mediating aSHM to about 1-2 kb downstream of the transcription start site [16] makes it difficult to distinguish bystander mutations from drivers on statistical grounds alone in the absence of mechanistic evidence of an impact on the transcription regulation of a candidate target of aSHM.

We addressed this difficulty in two ways. First, we analyzed *relative* enrichment of variants in the 5’ region of *CCND1* as compared to other types of lymphoma carrying the t(11;14)(q13;q32) translocation with evidence for mutated *IGHV*. To exclude variants selected at the level of protein function, we restricted our analysis to the set of non-coding variants. We observed MCL-specific enrichment of mutations in TFBSs. Spatial clustering of non-coding mutations at the *CCND1* locus in MCL samples has recently been reported by [15] using the tool OncodriveCLUST [41]. However, the mechanistic consequences of this clustering and the relative enrichment in MCL were not investigated in this study.

Second, we used motif searches to investigate the potential impact of clustered non-coding mutations on *CCND1* expression. While the largely overlapping motifs for TFBSs belonging to the same family make it difficult to pinpoint the precise factor bound at a specific site, a consistent picture seems to emerge. We find that both destructive and constructive mutations can be identified. Destructive mutations prevent transcription factor binding that would normally occur. We detected destructive mutations in MEF2, STAT, and HOX motifs. A sizable fraction of these mutations occurs in sequence contexts that are compatible with c-AID mediated mutagenesis, whereas nc-AID mediated mutagenesis did not seem to play a major role. MEF2 transcription factors can activate or repress transcription depending on their association with activating or repressive co-factors [42]. MEF2B mutations have been reported in mantle cell lymphoma [13]. *STAT6* mutations in the DNA binding region have been described in Hodgkin lymphoma [43]. HOX transcription factors are involved in T cell differentiation and are known as drivers of T-ALL [44, 45]. In mantle cell lymphoma, HOX genes are silenced by H3K27me3 chromatin methylation mediated by overexpression of EZH2 [46].

We also observed constructive mutations that change the binding specificity of a site or create new binding sites. NFAT-binding sites were created in five different locations in the first intron of *CCND1*. NFAT proteins have long been known as major targets of antigen receptors expressed on T and B cells [47]. Recently, elevated levels of NFATC1 have been observed in CLL caused by DNA hypomethylation of the *NFATC1* locus, and *NFATC1* expression levels were shown to correlate with higher expression of *CCND1* [48]. In the *CCND1* promoter region as well as in the first intron, we observe the creation of CEBP binding sites. CEBP is known to be a major activator of *CCND1* transcription [49]. Additionally, SOX binding sites are created in more than one location. *SOX11* has been recognized as a major oncogene in mantle cell lymphoma [1, 3, 50]. In the 5’UTR an E2F site is created in close proximity to a pre-existing E2F binding site. E2F transcription factors are major drivers of cell cycle progression and are activated by CCND1 expression in a self-regulatory loop via CCND1 mediated phosphorylation of RB1 [51]. Interestingly, we observed co-occurrence of two mutations in the first exon (11-69456186-G-A, 11-69456187-G-A) that also create an E2F binding site. Both variants are among the most frequently observed mutations in the *CCND1* locus. 11-69456186-G-A is a synonymous mutation while 11-69456187-G-A codes for E36K. The creation of an underlying E2F binding site may be an additional explanation for the frequently observed CCND1 E36K mutation. In support of this transcriptional perspective on the origin of E36K, we find that the Y44D mutation, which has been reported to increase CCND1 stability [11], is not a preferred mutation target in our MCL data set. Consistent with the view that in MCL the *CCND1* transcription level is increased by aSHM mediated mutagenesis, we observed more synonymous and benign amino acid changes in exon1 as compared to non-MCL samples with t(11;14)(q13;q32) translocation and mutated *IGHV*.

We observed statistically significant differential regulation of *CCND1* transcription depending on *CCND1* mutation status in 28 nnMCL cases present in our cohort. This number does not allow us to determine whether aberrations in the 3’UTR are mutually exclusive with aSHM-generated mutations in *CCND1*. Alternative polyadenylation [7], aberrations of miRNA target sites [6], or alternative splicing [52] could have an impact on *CCND1* mRNA levels. All samples used in this analysis are lymphomas and express *CCND1* mRNA at a sufficient level to sustain lymphomagenesis. This circumstance negatively influences the significance of aSHM-mediated upregulation of CCND1 expression levels. Furthermore, we have not been able to establish whether the *CCND1* mutation status has an impact on the severity of the disease. The utility of non-coding *CCND1* mutations as markers for the diagnosis of nnMCL is another open question.

In conclusion, we find that non-coding mutations in the *CCND1* locus are clustered to TFBSs in a MCL-specific manner. These mutations are associated with higher levels of *CCND1* transcript. Assuming that CCND1 activity is a rate-limiting factor in MCL lymphomagenesis, aSHM-generated mutations at the *CCND1* locus may be actively selected for their impact on transcription regulation. The selection process may be particularly pronounced in MCL, which could explain the abundance of non-coding *CCND1* mutations in this disease.

## Supplementary information

Supplementary table S1

Supplementary table S2

Supplementary table S3
